# Sequential Impact of Diabetes Mellitus on Deep Neck Infections: Comparison of the Clinical Characteristics of Patients with and without Diabetes Mellitus

**DOI:** 10.3390/healthcare12141383

**Published:** 2024-07-10

**Authors:** Ting-I Liao, Chia-Ying Ho, Shy-Chyi Chin, Yu-Chien Wang, Kai-Chieh Chan, Shih-Lung Chen

**Affiliations:** 1 School of Medicine, Chang Gung University, Taoyuan 33302, Taiwan; jacky2000891126@gmail.com (T.-I.L.); chiayingho23@gmail.com (C.-Y.H.); b25chin@cgmh.org.tw (S.-C.C.); kjchan5109@gmail.com (K.-C.C.); 2 Division of Chinese Internal Medicine, Center for Traditional Chinese Medicine, Linkou Chang Gung Memorial Hospital, Taoyuan 33305, Taiwan; 3 Department of Medical Imaging and Intervention, Linkou Chang Gung Memorial Hospital, Taoyuan 33305, Taiwan; 4 Department of Otorhinolaryngology & Head and Neck Surgery, New Taipei Municipal Tucheng Hospital, New Taipei City 23652, Taiwan; m7054@cgmh.org.tw; 5 Department of Otorhinolaryngology & Head and Neck Surgery, Linkou Chang Gung Memorial Hospital, Taoyuan 33305, Taiwan

**Keywords:** C-reactive protein, deep neck infection, diabetes mellitus, *Klebsiella pneumoniae*

## Abstract

Background: Deep neck infections (DNIs) can compromise the airway and are associated with high morbidity and mortality rates. Diabetes mellitus (DM) is a metabolic disorder characterized by chronic hyperglycemia that is associated with several comorbidities. We compared the clinical characteristics of DNI patients with and without DM. Methods: This study recorded the relevant clinical variables of 383 patients with DNIs between November 2016 and September 2022; of those patients, 147 (38.38%) had DM. The clinical factors between DNI patients with and without DM were assessed. Results: Patients with DM were older (*p* < 0.001), had higher white blood cell counts (*p* = 0.029) and C-reactive protein levels (CRP, *p* < 0.001), had a greater number of deep neck spaces (*p* = 0.002) compared to patients without DM, and had longer hospital stays (*p* < 0.001). *Klebsiella pneumoniae* was cultured more frequently from patients with DM than those without DM (*p* = 0.002). A higher CRP level (OR = 1.0094, 95% CI: 1.0047–1.0142, *p* < 0.001) was a significant independent risk factor for DM patients with prolonged hospitalization. The lengths of hospital stays in patients with poorly controlled DM were longer than those with well-controlled DM (*p* = 0.027). Conclusions: DNI disease severity and outcomes were worse in patients with DM than those without DM. Antibiotics effective against *Klebsiella pneumoniae* should be used for DNI patients with DM. DNI patients with DM and high CRP levels had more prolonged hospitalizations. Appropriate blood glucose control is essential for DNI patients with DM.

## 1. Introduction

A deep neck infection (DNI) is a lethal emergent bacterial infection of the deep neck spaces that can lead to cellulitis or abscess formation [1]. Although DNIs usually result from upper aerodigestive tract infections, such as odontogenic infections and pharyngotonsillitis [2,3,4], there is an increasing proportion of DNI cases with an unknown primary origin [5]. Treating DNIs is difficult because the infection is in close proximity to vital structures and the head and neck region has a complex anatomy [6]. Despite the availability of antibiotics, advances in diagnostic technology, and surgical debridement, DNIs are still capable of causing serious complications, including airway obstruction [7], and can spread to the mediastinum [2].

Patients with comorbidities, such as alcohol consumption, drug abuse, and diabetes mellitus (DM), are at increased risk of complications from DNIs [8]. DM affects almost 4% of the total population of Taiwan [9]. It is also the most common comorbidity in DNIs [10]. Animal and in vitro studies have found that hyperglycemia impairs the immune functions of hosts, including cellular immunity, complement activation, and neutrophil bactericidal function [11,12,13]. In turn, immune deficiency predisposes DM patients to invasive infections [14]. Multiple host factors in DM patients may increase the risk of skin and soft tissue infections, including uncontrolled hyperglycemia, disruption of the skin barrier, poor vascularization, and immune system dysfunction [15,16]. Uncontrolled hyperglycemia is the main culprit behind infection. Furthermore, the clinical course of DNIs differs between patients with and without DM [17,18]. The management of patients with a DNI and DM is complicated. In this study, we compared the clinical characteristics, outcomes, and prognosis of DNIs between patients with and without DM.

## 2. Materials and Methods

**Figure 1:**
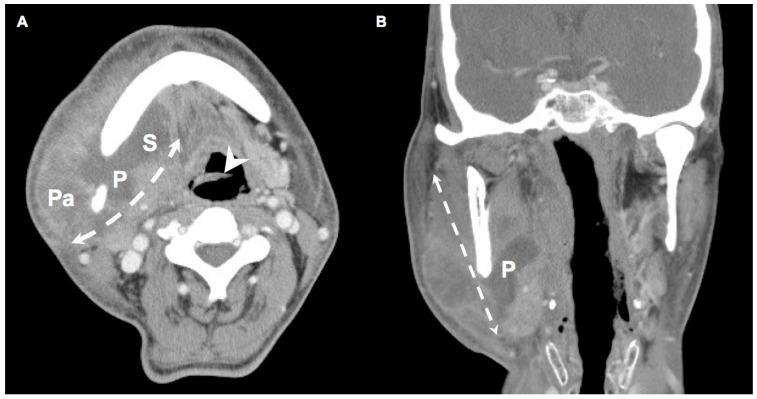
(**A**) Axial view and (**B**) coronal view of a DNI patient with DM. (**A**) indicates that the infection spread from the parapharyngeal space to the parotid space and the submandibular space, encompassing infection across multiple deep neck spaces. (**B**) depicts infection in the coronal plane. Arrowhead: epiglottis; Double dotted arrow: involved spaces of DNI; P: parapharyngeal space; Pa: parotid space; S: submandibular space. (300 × 300 dpi).

**Figure 2:**
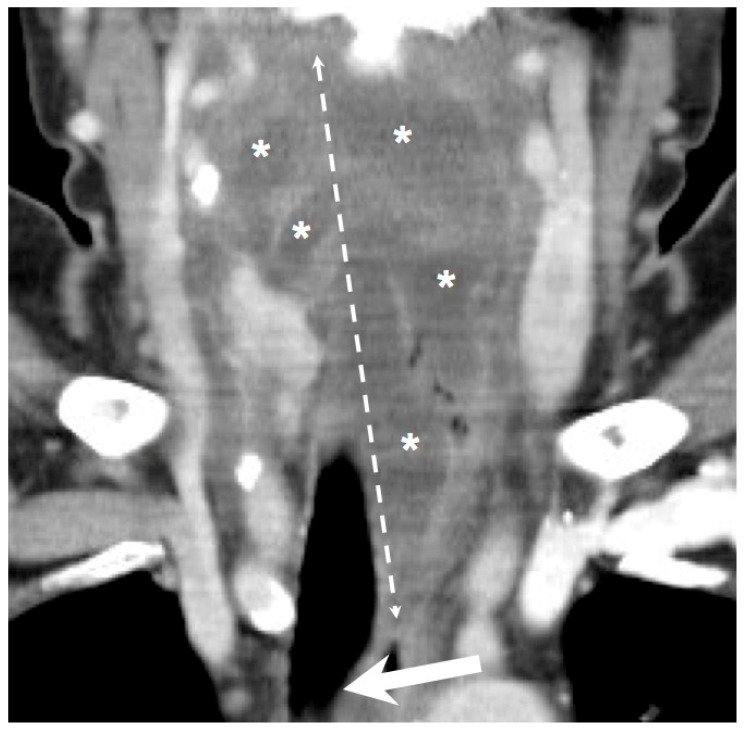
The coronal view showed extensive infection in the retropharyngeal spaces with poor DM control. Arrow: compromised and deviated airway; Asterisk; multiloculated deep neck abscess; Double dotted arrow: involved spaces of DNI. (300 × 300 dpi).

We retrospectively reviewed the medical records of 383 patients diagnosed with DNIs between November 2016 and September 2022 at the main branch of Chang Gung Memorial Hospital Linkou, a tertiary medical center in Taiwan. The patients were diagnosed on the basis of their clinical presentation and computed tomography (CT) findings (Figure 1 and Figure 2) [19]. The management of DNIs involved antibiotics and open surgical incision and drainage (I&D). Pathogen cultures were performed on specimens obtained using an aseptic procedure. Because primary culture results are often obtained after >3 days, empirical antibiotics were routinely administered prior to obtaining the culture results [20]. Ceftriaxone 1 gm q12h and metronidazole 500 mg q8h were used as empirical antibiotics for aerobic and anaerobic bacteria [17,21,22].

There were 147 (38.38%) and 236 (61.62%) patients with and without DM, respectively. All patients with DM were treated with appropriate anti-diabetic medications.

### 2.1. Exclusion Criteria

Patients with previous head and neck surgery, radiotherapy to the head and neck region, or accidental swallowing of a foreign body were excluded. In this article, we only enrolled patients with type II DM and excluded patients with type I DM. In total, 383 patients with DNIs were included, including 147 with DM.

### 2.2. Data Collection

We collected data on gender, age, chief complaint duration, length of hospital stays, white blood cell (WBC) count, C-reactive protein (CRP) level, duration of DM, involvement of multiple spaces (≥3), surgical I&D, tracheostomy, deep neck space involvement, mediastinitis, pneumonia, necrotizing fasciitis, septic shock, and identified pathogens.

### 2.3. Ethics Statement

This study was approved by the Institutional Review Board (IRB) of Chang Gung Medical Foundation (IRB no.: 202201624B0). The sample size of the study was not determined based on any criteria. We included patients based on IRB-approved enrollment time. Data were collected retrospectively and anonymized before the analysis. The IRB waived the requirement for informed consent from patients.

### 2.4. Statistical Analysis

The Kolmogorov–Smirnov test showed that the data were not normally distributed. Therefore, the chi-square test and Mann–Whitney *U* test were used to compare categorical and continuous variables, respectively. We performed univariate and multivariate analyses via logistic regression. We employed forward stepwise selection followed by multiple logistic regression; all variables included in the univariate analysis were entered into the final multivariable analysis. Data were analyzed using MedCalc software (version 18.6; MedCalc, Ostend, Belgium). *p* < 0.05 was considered to indicate statistical significance.

## 3. Results

Table 1 presents the clinical characteristics of the 383 patients with DNIs (254 [66.31%] males and 129 [33.69%] females; mean age: 52.01 ± 18.92 years). The mean chief complaint duration was 5.06 ± 4.34 days.

The mean WBC count was 15,360.65 ± 6041.59 µL and the mean CRP level was 134.67 ± 105.78 mg/L. There were 111 (28.98%) patients with involvement of multiple deep neck spaces (≥3).

The parapharyngeal, submandibular, retropharyngeal, parotid, masticator, anterior cervical, visceral, perivertebral, carotid, and posterior cervical spaces were involved in 230 (60.05%), 161 (42.03%), 109 (28.45%), 84 (21.93%), 74 (19.32%), 31 (8.09%), 22 (5.74%), 17 (4.43%), 12 (3.13%), and 8 (2.08%) patients, respectively.

Table 2 compares differences in the clinical characteristics of patients with and without DM. Among the individuals studied, there were 147 patients with DM and 236 patients without DM. In terms of age, there was a notable disparity between the two groups (*p* < 0.001); those with DM were considerably older, with a mean age of 56.28 ± 16.65 compared to 49.36 ± 19.77 for those without DM. When it comes to WBC and CRP, both levels were significantly higher in patients with DM (*p* = 0.029 and *p* < 0.001). The mean duration of DM was 3.93 ± 4.27 years, and mean HbA1c was 6.74 ± 0.51%. Regarding anti-diabetic drugs, 35 patients (23.81%) received insulin control, 85 patients (57.83%) used one anti-diabetic drug, and 27 patients (18.36%) used more than two anti-diabetic drugs. The proportion of more than three deep neck spaces involved in patients with DM is significantly higher than those without DM (38.09% vs. 23.31%, *p* = 0.002).

Table 3 illustrates the comparison of management strategies and morbidity outcomes between patients with and without DM. The mean length of hospital stay was 9.97 ± 8.38 days. The analysis indicated that the length of hospital stay in patients with DM was longer than those without DM (*p* < 0.001). Tracheostomy was performed in 47 (12.27%) patients, and 172 (44.91%) patients underwent open I&D for DNIs. Retinopathy, nephropathy, and neuropathy were noted in 17 (11.56%), 11 (7.48%), and 31 (21.08%) patients. Mediastinitis, pneumonia, necrotizing fasciitis, and septic shock were present in 25 (6.52%), 23 (6.01%), 19 (4.96%), and 17 (4.43%) patients, respectively. Nevertheless, there were no marked differences in the length of hospital days, number of tracheostomies, surgical I&D, morbidities, and mortality between patients with and without DM.

Table 4 shows the comparison of pathogens between patients with and without DM. The prevalence of *Klebsiella pneumoniae* between the two groups was significantly different (*p* = 0.002), and was higher in those with DM. *Klebsiella pneumoniae* (21.08%) was the most common pathogen in patients with DM, followed by *Streptococcus constellatus* (20.41%), *Parvimonas micra* (10.88%), and *Streptococcus anginosus* (10.88%). Meanwhile, *Parvimonas micra* (16.94%)*, Prevotella buccae* (15.67%), and *Streptococcus constellatus* (14.41%) were the top three most prevalent pathogens in patients without DM. 

Table 5 shows univariate and multivariate analyses of 147 DM patients for prolonged hospitalization. For univariate analysis, the higher WBC level (odds ratio (OR) = 1.0001, 95% confidence interval (CI): 1.0000–1.0002, *p* = 0.001), the higher CRP (OR = 1.0113, 95% CI: 1.0070–1.0156, *p* < 0.001), the involvement of at least three spaces (OR = 2.6625, 95% CI: 1.2889–5.4999, *p* = 0.007), and mediastinitis (OR = 4.3556, 95% CI: 1.3372–14.186, *p* = 0.013) were significant risk factors for prolonged hospitalization in DM patients. All factors were subjected to forward stepwise selection, which was followed by multivariate logistic regression. A higher CRP level (OR = 1.0094, 95% CI: 1.0047–1.0142, *p* < 0.001) was a significant independent risk factor for prolonged hospitalization.

Table 6 displays the comparison between DNI patients with poorly controlled (HbA1c ≥ 6.5) and well-controlled (HbA1c < 6.5) DM. The analysis indicated that the lengths of hospital stays in patients with poorly controlled DM were longer than those without DM (*p* = 0.027).

## 4. Discussion

Although DNIs are an emergent condition because of their severe sequelae, the mortality rate has significantly declined because of improved diagnostic techniques, greater availability of antibiotics, and the development of advanced surgical interventions [23,24]. Nevertheless, DNIs are still associated with severe complications and even death [2,6,20,21,25,26,27,28,29,30,31,32,33]. Thus, the treatment of DNIs remains challenging.

DM has a significant impact on the course of infections. In Taiwan, disease control using appropriate medications is achieved in 60% of DM patients; the remaining patients have uncontrolled disease [9]. DM represents a group of physiological dysfunctions characterized by hyperglycemia due to insufficient insulin production, glucagon hypersecretion (type 1 DM), or directly from insulin resistance (type 2 DM) [34,35]. Type 1 DM is a chronic autoimmune disease that affects about 1% of the population in developed countries [36]. This immune response is induced and promoted by the interaction of environmental as well as genetic factors [37]. In contrast, in type 2 diabetes, insulin resistance appears to be the primary cause of hyperglycemia (affecting around 8.5% of the adult population) [36]. Patients with systemic diseases such as DM may experience the opportunistic advancement of seemingly minor infections due to compromised immune systems and vascular function [18,38,39]. Huang et al. [1] found that the prevalence of DM was higher in DNI patients than in the general population of Taiwan. DM patients have a higher risk of infection and impaired cutaneous wound and soft tissue healing [40]. Thus, DNI patients with DM have more severe and extensive inflammation [41,42].

In the present study, DNI patients with DM were older and had longer hospital stays, a higher WBC count and CRP level, and a greater number of involved deep neck spaces compared to DNI patients without DM. Patients with DNI and DM have more serious infections, a worse prognosis, and a higher rate of *Klebsiella pneumoniae* infection compared to patients with DNI alone. 

In our research, patients with DM were older and had a longer hospital stay than patients without DM. Older patients with systemic diseases have impaired defense against pathogenic infections and a worse recovery rate, leading to prolonged hospital stays [10]. DM is an expected risk depending on the age of the patients. It is a risk factor that must be controlled in every surgery. In addition, DM patients also had a higher WBC counts and CRP levels, as well as greater numbers of involved deep neck spaces compared to patients without DM, suggesting that the DM patients had more severe inflammation and worse outcomes [43]. Sideris et al. [44] found that CRP level correlated with blood glucose level. Furthermore, the involvement of multiple deep neck spaces was a risk factor for the need for tracheostomy [45]. Cheng et al. [46] also found that DM patients were at an increased risk of necrotizing fasciitis. Based on the findings that DM patients have more severe DNIs and poor outcomes, blood glucose levels should be monitored and appropriately controlled.

Timely open surgery is essential for the treatment of DNIs, and repeated surgical I&D is necessary in some patients [47]. Therapeutic needle aspiration has also been performed in select cases [10,48]. Although pus cultures obtained via needle aspiration or surgical drainage can aid the selection of appropriate antibiotics, empirical antibiotics should be administered before the culture results become available [49].

A DNI is typically caused by polymicrobial aerobic and anaerobic organisms [50]. Studies from different countries and areas have reported different pathogenic causes of DNIs [21,51]. Furthermore, the causative organisms of DNIs may change over time and might be influenced by the choice of antibiotics [20].

We found that DM patients had a higher rate of *Klebsiella pneumoniae* infection than patients without DM, in line with previous studies [10,18]. One study found that *Klebsiella pneumoniae* is responsible for more than half of all DNI cases among DM patients [1]. *Klebsiella pneumoniae* is a Gram-negative aerobe [20]. The higher rate of identification of *Klebsiella pneumoniae* in DNI patients with DM may be because of oropharyngeal colonization by Gram-negative bacilli and a defective host defense, particularly phagocytosis [52,53]. Although odontogenic infection is the most common etiology of DNIs [24,54,55], Huang et al. [49] found that DM patients had a high culture rate of *Klebsiella pneumoniae*, irrespective of the odontogenic or nonodontogenic origin of their DNI. *Klebsiella pneumoniae* is susceptible to cefazolin, gentamicin, and trimethoprim/sulfamethoxazole [21]. However, clindamycin should not be administered alone because of its weak effect against *Klebsiella pneumoniae* [49].

Uncontrolled hyperglycemia correlates with a fatal outcome in DM patients with infectious diseases [56]. Appropriate blood glucose control reduces the microangiopathic and macroangiopathic complications of DM [57,58]. To achieve a favorable prognosis, the blood glucose level should be maintained below 200 mg/dL [1,10]. For optimal empiric coverage, it is recommended to use either penicillin in combination with a *β*-lactamase inhibitor or a *β*-lactamase–resistant antibiotic combined with a drug highly effective against most anaerobes [59,60]. Moreover, the addition of gentamicin is for effective Gram-negative coverage against *Klebsiella pneumoniae*, especially in DM patients, when it is resistant to clindamycin [55].

## 5. Study Limitations

This was a retrospective study performed at a single tertiary hospital. The treatments and management for patients with DNIs may have been relatively similar. When performing research in the future, patients could be enrolled from multiple hospitals to reduce such biases. Additionally, our study included only 383 cases. An increased number of samples can provide more comprehensive results.

Ideally, patients with infections should be switched from oral hypoglycemic agents to insulin. However, some patients still want to maintain their original oral hypoglycemic agents for treatment during hospitalization. As long as the patient’s condition did not worsen, we kept the patient’s original oral hypoglycemic agents.

Diabetes complications, such as diabetic retinopathy and peripheral neuropathy, can lead to balance impairment [61]. However, this retrospective article mainly discusses the impact of DM on DNI patients with potential risk of mortality and does not document whether the patients suffered from diabetic balance or vertigo. The effect of DM on balance is an important topic worthy of future research.

## 6. Conclusions

Our data showed several characteristic features among DM patients with DNIs, including an older age, elevated levels of WBC and CRP, the involvement of multiple spaces, and extended hospital stays. Therefore, we recommend a more rigorous approach when dealing with DNIs in DM patients, emphasizing the importance of good glycemic control to prevent the occurrence of more severe complications. In addition, since the prevalence of *Klebsiella pneumoniae* is increased in patients with DM, the selection of empiric antibiotics should also take this into consideration.

## Figures and Tables

**Table 1:** Clinical characteristics of the 383 patients with DNIs.

Characteristics	N (%)
Gender	383 (100.0)
Male	254 (66.31)
Female	129 (33.69)
Age, years ± SD	52.01 ± 18.92
Chief complaint duration, days ± SD	5.06 ± 4.34
WBC, µL ± SD	15,360.65 ± 6041.59
CRP, mg/L ± SD	134.67 ± 105.78
Multiple spaces involved, ≥3	111 (28.98)
Deep neck space involvement	
Parapharyngeal space	230 (60.05)
Submandibular space	161 (42.03)
Retropharyngeal space	109 (28.45)
Parotid space	84 (21.93)
Masticator space	74 (19.32)
Anterior cervical space	31 (8.09)
Visceral space	22 (5.74)
Perivertebral space	17 (4.43)
Carotid space	12 (3.13)
Posterior cervical space	8 (2.08)

DNI = deep neck infection; N = numbers; SD = standard deviation; WBC = white blood cell (normal range: 3900–10,600/µL); CRP = C-reactive protein (normal range < 5 mg/L).

**Table 2:** Comparison of clinical characteristics between patients with and without DM.

Characteristics	DM; N = 147 (%)	Non-DM; N = 236 (%)	*p*-Value
Gender	147 (100.0)	236 (100.0)	
Male	94 (63.94)	160 (67.79)	0.439
Female	53 (36.06)	76 (32.21)	
Age, years ± SD (median; min, max)	56.28 ± 16.65 (56; 93, 19)	49.36 ± 19.77 (49.5; 98, 20)	**<0.001**
Chief complaint duration, days ± SD (median; max, min)	5.35 ± 4.71 (4; 30, 1)	4.87 ± 4.11 (4; 30, 2)	0.382
WBC, µL ± SD (median; max, min)	16,017.89 ± 6124.39 (15,800; 32,900, 2000)	14,951.26 ± 5965.88 (13,700; 42,700, 4100)	**0.029**
CRP, mg/L ± SD (median; max, min)	166.76 ± 109.95 (163; 480, 27)	124.29 ± 99.91 (105; 487, 21)	**<0.001**
Duration of DM, years	3.93 ± 4.27		
HbA1c, %	6.74 ± 0.51		
On insulin control	35 (23.81)		
On one anti-diabetic drug	85 (57.83)		
On more than two anti-diabetic drugs	27 (18.36)		
Multiple spaces involved, ≥3			**0.002**
Yes	56 (38.09)	55 (23.31)	
No	91 (61.91)	181 (76.69)	
Parapharyngeal space			0.453
Yes	92 (62.58)	138 (58.47)	
No	55 (37.42)	98 (41.53)	
Submandibular space			0.670
Yes	64 (43.53)	97 (41.11)	
No	83 (56.47)	139 (58.89)	
Retropharyngeal space			0.103
Yes	49 (33.33)	60 (25.42)	
No	98 (66.67)	176 (74.58)	
Parotid space			0.374
Yes	36 (24.48)	48 (20.33)	
No	111 (75.52)	188 (79.67)	
Masticator space			0.507
Yes	31 (21.08)	43 (18.22)	
No	116 (78.92)	193 (81.78)	
Anterior cervical space			1.000
Yes	12 (8.16)	19 (8.05)	
No	135 (91.84)	217 (91.95)	
Visceral space			0.504
Yes	10 (6.81)	12 (5.08)	
No	137 (93.19)	224 (94.92)	
Perivertebral space			0.455
Yes	8 (5.44)	9 (3.81)	
No	139 (94.56)	227 (96.19)	
Carotid space			0.225
Yes	7 (4.76)	5 (2.11)	
No	140 (95.24)	231 (97.89)	
Posterior cervical space			0.268
Yes	5 (3.41)	3 (1.27)	
No	142 (96.59)	233 (98.73)	

CRP = C-reactive protein (normal range < 5mg/L); DM = diabetes mellitus; N = numbers; SD = standard deviation; WBC = white blood cell (normal range: 3900–10,600/µL); Significant differences with *p* < 0.05 are shown in bold.

**Table 3:** Comparison of management and morbidity between patients with and without DM.

Characteristics	All; N = 383(%)	DM; N = 147 (%)	Non-DM; N = 236 (%)	*p*-Value
Length of hospital stay, days ± SD (median; max, min)	9.97 ± 8.38 (8; 52, 2)	11.69 ± 9.14 (9; 52, 4)	8.91 ± 7.69 (7; 51, 2)	**<0.001**
Tracheostomy				0.077
Yes	47 (12.27)	24 (16.32)	23 (9.74)	
No	336 (87.73)	123 (83.68)	213 (90.26)	
Surgical incision and drainage				0.245
Yes	172 (44.91)	72 (48.97)	100 (42.37)	
No	211 (55.09)	75 (51.03)	136 (57.63)	
Retinopathy		17 (11.56%)		
Nephropathy		11 (7.48%)		
Neuropathy		31 (21.08%)		
Morbidity				
Mediastinitis	25 (6.52)	13 (8.84)	12 (5.08)	0.201
Pneumonia	23 (6.01)	10 (6.81)	13 (5.51)	0.661
Necrotizing fasciitis	19 (4.96)	11 (7.48)	8 (3.38)	0.091
Septic shock	17 (4.43)	8 (5.44)	9 (3.81)	0.455
Mortality	29 (7.57)	16 (10.88)	13 (5.51)	0.072

DM = diabetes mellitus; N = number; *p* < 0.05. Significant differences are shown in bold.

**Table 4:** Comparison of pathogens between patients with and without DM.

Pathogens	All, N = 383 (%)	DM; N = 147 (%)	Non-DM; N = 236 (%)	*p*-Value
*Streptococcus constellatus*	64 (16.71)	30 (20.41)	34 (14.41)	0.158
*Parvimonas micra*	56 (14.62)	16 (10.88)	40 (16.94)	0.136
*Klebsiella pneumoniae*	54 (14.09)	31 (21.08)	23 (9.74)	**0.002**
*Prevotella buccae*	50 (13.05)	13 (8.84)	37 (15.67)	0.061
*Streptococcus anginosus*	40 (10.44)	16 (10.88)	24 (10.16)	0.864
*Prevotella intermedia*	32 (8.35)	10 (6.81)	22 (9.32)	0.451
*Staphylococcus aureus*	21 (5.48)	8 (5.44)	13 (5.51)	1.000
*Staphylococcus epidermidis*	17 (4.43)	10 (6.81)	7 (2.96)	0.123
*Streptococcus oralis*	11 (2.87)	7 (4.76)	4 (1.69)	0.114
*Eikenella corrodens*	11 (2.87)	5 (3.41)	6 (2.54)	0.755
*Streptococcus salivarius*	11 (2.87)	4 (2.72)	7 (2.96)	1.000
*Gemella morbillorum*	8 (2.08)	5 (3.41)	3 (1.27)	0.268
*Slackia exigua*	7 (1.82)	4 (2.72)	3 (1.27)	0.435
*Pseudomonas aeruginosa*	6 (1.56)	1 (0.68)	5 (2.11)	0.413
*Salmonella enterica*	4 (1.04)	3 (2.04)	1 (0.42)	0.159
No growth	47 (12.27)	13 (8.84)	34 (14.41)	0.112

DM = diabetes mellitus; N = number. Significant differences with *p* < 0.05 are shown in bold.

**Table 5:** Univariate and multivariate analyses of 147 DM patients for prolonged hospitalization.

Variable	Prolonged Hospitalization		Univariate Analysis			Multivariate Analysis		
	Yes	No	OR	95% CI	*p*-Value	OR	95% CI	*p*-Value
Gender	44	103	1.3451	0.6506–2.7806	0.423			
Yes	26	68						
No	18	35						
Age, years ± SD	59.79 ± 18.79	54.78 ± 15.51	1.0188	0.9967–1.0414	0.096			
WBC, µL ± SD	18,547.71 ± 6508.69	142,937.18 ± 5647.04	1.0001	1.0000–1.0002	**0.001**	-	-	-
CRP, mg/L ± SD	247.11 ± 96.28	132.44 ± 97.08	1.0113	1.0070–1.0156	**<0.001**	1.0094	1.0047–1.0142	**<0.001**
Multiple spaces, ≥3					**0.007**	-	-	-
Yes	24	32	2.6625	1.2889–5.4999				
No	20	71	1.0000					
Mediastinitis					**0.013**	-	-	-
Yes	8	5	4.3556	1.3372–14.186				
No	36	98	1.0000					

CI = confidence intervals; CRP = C-reactive protein; DM = diabetes mellitus; OR = odds ratio; SD = standard deviation; WBC = white blood cell; *p* < 0.05. Significant differences are shown in bold.

**Table 6:** Comparison between DNI patients with poorly controlled and well-controlled DM.

Characteristics	Poorly Controlled (HbA1c ≥ 6.5)	Well-Controlled (HbA1c < 6.5)	*p*-Value
Number	90	57	
Length of hospital stay, days ± SD (median; max, min)	12.59 ± 8.82 (11; 52, 4)	10.29 ± 9.84 (7; 51, 2)	**0.027**
WBC, µL ± SD (median; max, min)	15,832.21 ± 6321.81 (15,550; 32,900, 2000)	16,311.11 ± 5842.13 (16,100; 42,700, 5400)	0.576
CRP, mg/L ± SD (median; max, min)	171.53 ± 116.83 (157.5; 480, 27)	159.23 ± 98.65 (166; 487, 21)	0.631

CRP = C-reactive protein; DNI = deep neck infection; DM = diabetes mellitus; WBC = white blood cell; *p* < 0.05. Significant differences are shown in bold.

## Data Availability

Data are contained within the article.

## Abbreviations

CT: Computed tomography; CRP: C-reactive protein; DNI: Deep neck infection; DM: Diabetes mellitus.
